# Using Health Care Utilization and Publication Patterns to Characterize the Research Portfolio and to Plan Future Research Investments

**DOI:** 10.1371/journal.pone.0114873

**Published:** 2014-12-10

**Authors:** Luba Katz, Rebecca V. Fink, Samuel R. Bozeman, Barbara J. McNeil

**Affiliations:** 1 US Health Division, Abt Associates Inc., Cambridge, Massachusetts, United States of America; 2 Department of Health Care Policy, Harvard Medical School, Boston, Massachusetts, United States of America; Universidad Veracruzana, Mexico

## Abstract

**Objective:**

Government funders of biomedical research are under increasing pressure to demonstrate societal benefits of their investments. A number of published studies attempted to correlate research funding levels with the societal burden for various diseases, with mixed results. We examined whether research funded by the Department of Veterans Affairs (VA) is well aligned with current and projected veterans’ health needs. The organizational structure of the VA makes it a particularly suitable setting for examining these questions.

**Methods:**

We used the publication patterns and dollar expenditures of VA-funded researchers to characterize the VA research portfolio by disease. We used health care utilization data from the VA for the same diseases to define veterans’ health needs. We then measured the level of correlation between the two and identified disease groups that were under- or over-represented in the research portfolio relative to disease expenditures. Finally, we used historic health care utilization trends combined with demographic projections to identify diseases and conditions that are increasing in costs and/or patient volume and consequently represent potential targets for future research investments.

**Results:**

We found a significant correlation between research volume/expenditures and health utilization. Some disease groups were slightly under- or over-represented, but these deviations were relatively small. Diseases and conditions with the increasing utilization trend at the VA included hypertension, hypercholesterolemia, diabetes, hearing loss, sleeping disorders, complications of pregnancy, and several mental disorders.

**Conclusions:**

Research investments at the VA are well aligned with veteran health needs. The VA can continue to meet these needs by supporting research on the diseases and conditions with a growing number of patients, costs of care, or both. Our approach can be used by other funders of disease research to characterize their portfolios and to plan research investments.

## Introduction

### Establishing a link between research expenditures and value to society

In the current climate of fiscal austerity, research funders in the United States and in other countries are under increasing pressure from their governments to demonstrate societal benefits of research investments [1]. A number of published studies report a correlation between research spending and disease burden, which is commonly used as a proxy measure for societal need [2], [3], [4], [5], [6]. Various indicators for disease burden include years of life lost, disability-adjusted life years, mortality rates, disease incidence and prevalence, number of patient visits and hospital days, and health care expenditures.

These studies found highly variable correlation between funding and burden. For example, Rubin and colleagues compared the number of projects funded by the National Institute of Biomedical Imaging and Bioengineering (NIBIB) at the National Institutes of Health (NIH) to three measures of disease burden, including cause of death, disability-adjusted survival losses, and health expenditures [3]. While the study revealed a positive correlation between research investments and disease burden, the number of research projects in cancer and heart disease was too high and in injuries/trauma, mental health, and respiratory diseases too low, given the burden of these diseases. Carter *et al* carried out a similar study for the National Cancer Institute at NIH and observed significant mismatches in the expenditures and burden for different types of cancer [7]. Gillum and colleagues analyzed 2006 funding data at NIH for 29 common diseases and found that the levels of research funding weakly correlated with disease burden, and that the correlation has not strengthened over time [8]. Studies comparing funding levels and burden for a range of diseases in Canada, the United Kingdom, Australia, and Spain produced similar results [9], [10], [11], [12].

At least two factors may explain why a stronger positive correlative relationship between funding levels and disease burden was not observed. First, these studies typically considered only research expenditures from a single funder, for example the NIH in the United States [2], [3], [5], [8] or the National Health and Medical Research Council in Australia [4], [7]. This approach would likely underestimate the levels of research funding, as many biomedical scientists receive support from several sources, including local government, non-profit foundations, and the pharmaceutical industry [13]. In addition, portfolios of many funders include large investments in basic research, which can be difficult to link to disease burden. At NIH, for example, basic research expenditures account for 54% of the budget [14]. Consequently, Rubin and colleagues could not classify roughly half of all NIBIB projects because they were not focused on specific diseases [3]. A recent study conducted by Sampat and colleagues concluded that NIH programs focusing on more applied research were better aligned with disease burden estimates [15].

We examined the relationship between the composition of the medical research portfolio and the burden of disease at the Department of Veterans Affairs (VA). The VA represents an especially suitable study setting because its research and health care are contained within the same organization and because the VA research is largely disease focused and thus is easier to link to health expenditures. In addition to correlating research volume and health care utilization at present, we analyzed possible future veteran health needs using population and expenditure trends.

### VA Medical Research Program

The mission of the VA medical research program is to “discover knowledge, develop VA researchers and health care leaders, and create innovations that advance health care for our Veterans and the Nation” [16]. The VA medical research program consists of a network of intramural investigators based at 109 medical centers across the United States. Support for the program comes from three principal sources: the President’s budget for VA Medical and Prosthetics Research ($581 million in 2010), the Veterans Equitable Resource Allocation system ($453 million in 2010), and other federal and non-federal agencies and entities ($727 million in 2010), in particular the NIH and the Department of Defense [17]. Funding for veteran health care comes from a different portion of the federal budget and these funding streams are non-overlapping.

The research program is administered by the Office of Research and Development, which allocates VA funding via several mechanisms, including research awards (similar to the NIH R01 series), career development awards (similar to the NIH K award series), and research centers of excellence (similar to the NIH P series) [17].

The Office of Research and Development seeks to shape the research portfolio by issuing funding announcements in particular areas and/or using one of several funding mechanisms. However, the selection of proposals for funding is ultimately based on the results of a peer review process, whereby a panel of experts in the applicants’ fields evaluates each proposal based on its significance, scientific approach, feasibility, innovation, and relevance to health needs of veterans [18].

Research funding is allocated to the VA community via four services: Biomedical (37% of researchers), Clinical (23% of researchers), Health Services (24% of researchers), and Rehabilitation (16% of researchers). Research in the Biomedical service emphasizes pre-clinical development: two-thirds of the projects use human tissues and the remaining use animal models (personal communication with the VA leadership). The Clinical service supports human subject research to determine the feasibility and effectiveness of new treatments and interventions. Health services research focuses on various aspects of health care delivery at the VA. Finally, the Rehabilitation service conducts research in the areas of tissue engineering, prosthetics, orthotics, and other assistive devices.

Approximately 3,300 individuals eligible to receive intramural funding constitute the core of the VA research community. An additional 8,000 researchers are affiliated with the VA and have access to VA facilities, resources, or patients, but do not receive support from the Office of Research and Development. Only a subset of physicians at the VA is involved in research: in 2008 the total number of physicians was estimated at 15,175 [19], compared to roughly 3,000 conducting research.

The VA research program is particularly well suited to explore the relationship between research expenditures and population needs for several reasons. First, the program is intramural - open only to the researchers employed at the VA. As a result, the research community is relatively stable and variation in portfolio composition from year to year is small [17]. Second, the VA provides health care to veterans and their families and systematically collects data on health care expenditures, making utilization analysis possible. Third, VA research is mostly clinical and, therefore, easier to link to disease burden. Finally, roughly 70% of VA-funded researchers are also physicians caring for veterans, forging a more direct connection between research and clinical duties [20].

Our study had two goals: to examine whether the VA research portfolio aligns with veteran health needs at present and to identify targets for future research investments based on the demographic and health care utilization trends.

## Methods

### Determination of Disease Burden

Utilization of VA health services, including dollar expenditures and patient counts, was used as a measure of disease burden. The VA provided three datasets: (1) aggregate cost, number of patients, and patient visit count data sorted by International Statistical Classifications of Diseases Version 9 (ICD-9) codes for 2006–2011; (2) enrollments in the VA health care from 2010 projected to 2040 by age and gender; and (3) patient counts by age, gender, and ICD-9 code for 2009–2011.

The ICD-9 dataset contained 1,024 codes grouped into disease categories as shown in Table 1. We combined a small group of diseases of the blood and blood-forming organs (expenditures of $231.8 M in 2010) with a much larger group of diseases of circulatory system ($4.4B) for more robust analyses. Inpatient and outpatient expenditures for each ICD-9 code were combined to determine total expenditures. We stratified ICD-9 - sorted patient counts by age and gender to identify diagnoses with relatively high proportions of elderly or female beneficiaries. Patient counts and dollar expenditures were also used to identify diagnoses with large increases over a five-year period.

**Table 1:** Healthcare expenditures by ICD-9 code group and research volume by number of PIs for 2010.

Disease or condition(ICD-9 codes)	Number of uniquepatients(percent total)	Dollarexpenditures(percent total)	Numberof visits(percent total)	Numberof PIs(percent total)
Infectious andparasitic diseases(001–139)	434,348 (2)	592,198,445 (2)	733,529 (1)	102 (6)
Neoplasms(140–239)	770,927 (4)	2,258,032,989 (8)	2,239,762 (4)	147 (9)
Endocrine, nutritional,metabolic, andimmune diseases(240–279)	2,253,612 (11)	1,497,467,562 (6)	7,676,148 (15)	117 (7)
Mentaldisorders (290–319)	2,354,690 (12)	5,398,542,344 (20)	12,334,042 (24)	320 (20)
Diseases of the nervoussystem and sense organs(320–389)	2,843,982 (14)	2,238,004,578 (8)	5,554,221 (11)	226 (14)
Diseases of thecirculatory system,blood, and blood-formingorgans (280–289/390–459)	3,391,518 (17)	4,638,191,610 (17)	6,860,838 (14)	210 (13)
Diseases of therespiratory system (460–519)	1,075,092 (5)	1,714,401,907 (6)	1,741,168 (3)	74 (5)
Diseases of thedigestive system (520–579)	1,441,085 (7)	2,081,529,854 (8)	2,356,636 (5)	75 (5)
Diseases of thegenitourinarysystem (580–629)	787,936 (4)	1,747,570,592 (7)	2,390,704 (5)	86 (5)
Complications of pregnancyand childbirth/congenitalabnormalities/conditionsoriginating in the perinatalperiod(630–676/740–759/760–779)	50,552 (0)	52,236,518 (0)	78,406 (0)	0 (0)
Diseases of theskin and subcutaneoustissue (680–709)	852,777 (4)	723,281,237 (3)	1,364,251 (3)	26 (2)
Diseases of the musculoskeletalsystem and connectivetissue (710–739)	2,747,423 (14)	2,373,896,550 (9)	5,719,424 (11)	76 (5)
Injury and poisoning(800–999/E80–E99)	731,800 (4)	1,407,110,520 (5)	1,501,366 (3)	151 (9)
Total	19,735,742	26,722,464,706	50,550,495	1,610

### Classification of Research Activities

We used two approaches to classify the VA research portfolio. First, we examined publication patterns of the VA-funded Principal Investigators (PIs) to estimate research volume for each disease group. We queried Web of Knowledge, a publically available publication database, using PI names and affiliations. We included all PIs with active VA grants in 2010 in the search. Web of Knowledge automatically assigns each article to one or more of the 250 research categories based on the focus of the journal in which the article was published [21]. Using these categories as a guide, we assigned every PI to a single disease group. For PIs who published articles across multiple disease groups, we assigned the category with the most articles. If the number of articles was evenly split between research categories, we assigned the category with the largest number of publications citing VA as a funder.

Note that at VA the size of the grants is capped at $250,000 and only one grant is awarded per PI per service. While it is possible that the same researcher is funded simultaneously by two or more services, it is not common. Therefore, the number of PIs working on a disease closely mirrors the actual VA research investments on this disease. Data on non-VA funding per PI were not available from VA.

In addition, the VA provided an incomplete research expenditure dataset for 2010 with aggregate research dollar expenditures for the following disease areas: cancer, infectious, endocrine, metabolic, immunity, mental, nervous, circulatory, respiratory, digestive, genitourinary, and muscular-skeletal diseases. No data were available for injury, poisoning, pregnancy/childbirth, and skin diseases.

### Statistical Analysis

We calculated a Pearson correlation statistic to analyze the relationship between research volume, expressed as number of PIs and as dollar amount spent on research, and healthcare utilization, expressed as health expenditures, number of patient visits, and number of patients for 2010. Simple linear regression modelling was used to predict research volume based on disease expenditures. All calculations were performed with SAS/STAT Version 9.3 for Windows, 2010 (SAS Institute, Cary, North Carolina).

### Ethics Statement

The Abt Associates Institutional Review Board determined that this research study did not involve human subjects. Informed consent was not given to participants for their clinical records to be used in this study. Patient information was anonymized and de-identified prior to analysis.

## Results

### Data Loss

We excluded some data because of classification uncertainty. Of 1,967 PIs in the sample, 286 (15%) could not be linked to a disease category because they either did not work on diseases, had no publications in Web of Knowledge, or both. For example, we could not assign research on basic cellular processes or on developing imaging tools. For health care expenditures, ICD-9 codes V01-V89 were excluded because they are used to document general factors influencing health status and cannot be mapped onto a specific disease group. This exclusion resulted in the loss of 17% of health expenditure data.

### Research Portfolio and Disease Burden

Total health care expenditures by disease group at VA in 2010 are shown in Table 1. Mental disorders and diseases of the circulatory system emerged as the most costly diagnostic categories, together accounting for 37% of all expenditures, 38% of all patient visits, and 29% of all patients. Table 1 also shows the number of VA-funded PIs publishing in each of the disease categories. The number of PIs ranged from a high of 320 PIs or 20% of all investigators for mental disorders to a low of 76 PIs for diseases of the digestive system, representing 5% of all investigators. At the time of this study VA did not fund research on pregnancy and related disorders.

Table 2 compares the VA research portfolio to the burden of diseases on the VA health care system. Two measures of research investment and three measures of health expenditures were used in the calculations. Research volume expressed as the number of PIs was highly correlated with all three measures of health utilization, and research volume expressed as dollar expenditures was highly correlated with two measures of health utilization. Only the correlation between the number of patients and research investment in dollars was non-significant (Table 2).

**Table 2:** Correlation between research volume and health care utilization at VA in 2010.

Researchvolume	Healthcare dollarexpenditures r (p-value)	Number of uniquepatients r (p-value)	Number ofvisits r (p-value)
Number of PIs	0.83 (0.0004)	0.61 (0.0272)	0.78 (0.0016)
Researchexpenditures(dollars)	0.78 (0.0083)	0.46 (0.1787)	0.81 (0.0044)

Pearson correlation is shown.

**Figure 1:**
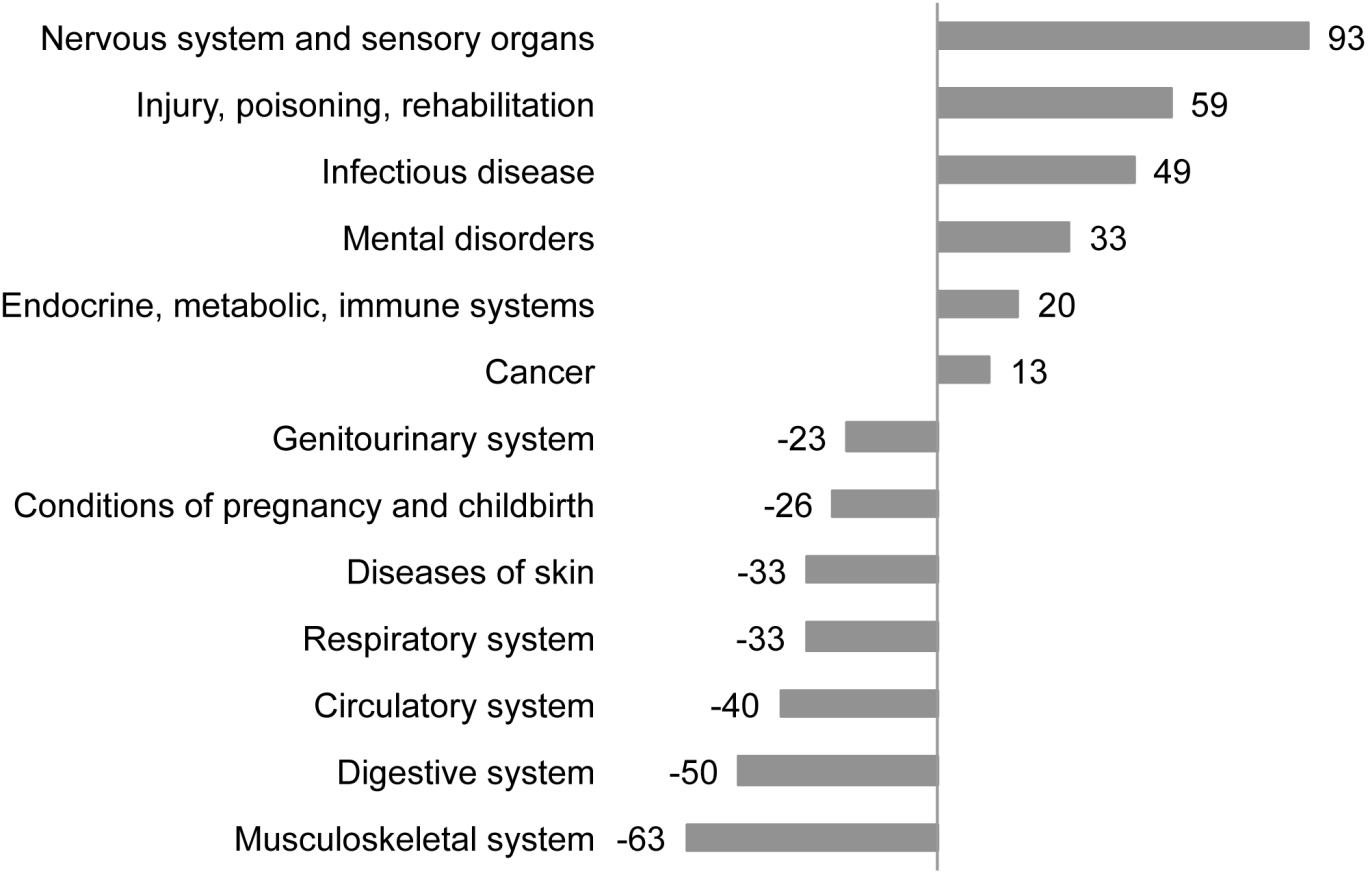
Difference between predicted and actual number of projects, given health care expenditures in 2010. Simple linear regression modelling was used to predict research volume for each disease group.

Fig. 1 shows the differences between the number of PIs observed for each disease group in the actual data and the number of PIs predicted from our linear model regressing the number of PIs on the dollars expended by disease category. The true number of PIs publishing on the diseases of the nervous system, injury/rehabilitation, and infectious disease was somewhat higher than expected and the number of PIs publishing on the musculoskeletal, digestive, and circulatory systems lower than expected.

### Future Health Needs of Veterans

**Figure 2:**
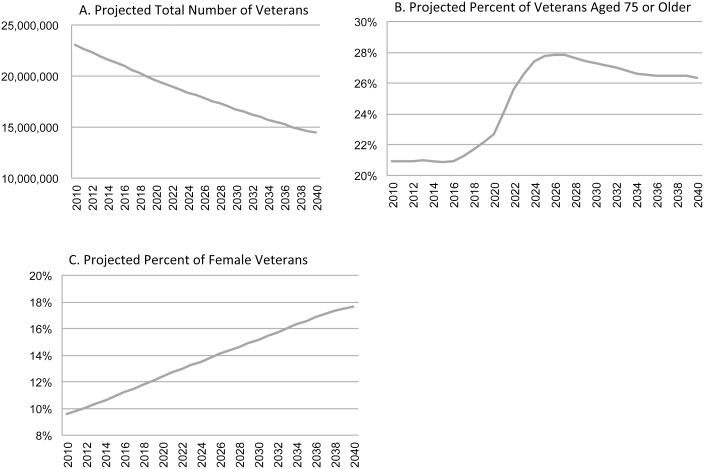
Demographic projections for veteran enrollment in VA health care. Data provided by the Department of Veterans Affairs.

Enrollment in VA health care is expected to decline over the next 20 years, from 23 million in 2010 to an estimated 14 million in 2040 (Fig. 2). However, the proportion of enrolled veterans aged 75 or over is expected to increase from 21% to 26% and the proportion of enrolled female veterans from 10% in 2010 to 18% in 2040. Other factors unchanged, these population shifts predict increases in patient volume and costs for diseases and conditions that disproportionately affect these patient groups.

We examined the VA ICD-9 data for 2009–2011 to identify the codes with the largest average number of patients in these demographic categories (health care expenditure data by age and gender were unavailable). Large numbers of both women and elderly veterans sought care for hypertension, hypercholesterolemia, and adjustment reaction (Table 3). The VA treated many elderly veterans for diabetes and hearing loss, and many women for affective psychoses and depression.

**Table 3:** Diseases with highest prevalence among elderly or female patients.

Disease	Number of patients75 years and older	Number offemale patients	Disease group
Hypertension	1,587,809	66,777	Circulatory system
Diabetes mellitus	999,194		Endocrine system
Hypercholesterolemia	711,257	38,725	Endocrine system
Adjustment reaction	541,110	45,701	Mental disorders
Conductive hearing loss	530,999		Nervous system
Affective psychoses		48,161	Mental disorders
Depressive disorder		42,393	Mental disorder

Average for 2006–2009 is shown.

We also projected future costs of care at VA by identifying the diseases and conditions with recent large increases in utilization. Table 4 shows ICD-9 codes with at least a 100% increase in cost and a 50% increase in the number of patients between 2006 and 2011. Complications of pregnancy, organic sleep disorders, nephrotic conditions, and endocrine gland conditions have increased both in cost and patient counts. In addition, arthropathies (inflammation of the joints) and dorsopathies (diseases of the spine) increased in cost and post-traumatic stress disorder and bipolar disorder in patient counts.

**Table 4:** Diseases and conditions that increased by 100% or more in cost or by 50% or more in patient volume between 2006 and 2011.

Disease orcondition	Percent increasein cost	Percent increase innumber of patients	Diseasegroup
Complications ofpregnancy	622	60	Complications of pregnancy
Organic sleepdisorders	379	367	Nervous system
Nephroticconditions	145	53	Genitourinary system
Endocrinegland diseases	115	69	Endocrine system
Arthropathies	108		Musculoskeletal system
Dorsopathies	101		Musculoskeletal system
Post-traumaticstress disorder		69	Mental disorders
Bipolar disorder		50	Mental disorders

## Discussion

We investigated the relationship between the composition of the VA disease research portfolio and health needs of veterans using several different measures and with one exception found a significant, positive correlation. Some disease categories appeared to be over- or under-represented in the VA research portfolio relative to the costs of care, but these deviations were relatively small.

We also used ICD-9 and demographic data to identify diseases and conditions that are likely to be costly to the VA health system in the future. Demographic projections suggest that the number of elderly and women veterans enrolled in VA health care is expected to increase in the next 20 years. Three conditions – adjustment reaction, hypertension, and hypercholesterolemia – were prevalent among both elderly and women patients. We also identified several diseases and conditions that have significantly increased in cost and/or patient volume since 2006. These included complications of pregnancy, sleep disorder, post-traumatic stress disorder, endocrine gland disease, nephrotic conditions, bipolar disorder, and two musculoskeletal diseases. Based on these data, we argue that the VA should sustain or increase its research investments in some or all of these disease areas. Better treatment options resulting from more research may increase the costs of care. However, improved quality of life and/or life expectancy for veteran patients and for the general public are valuable outcomes of research investment, regardless of costs.

As pointed out by others [8], [9], it is not feasible to allocate research funding solely based on the burden of disease. The VA and other federal agencies must balance multiple factors and inputs from a range of stakeholders in making investment decisions. For example, research in injury and rehabilitation – which is overrepresented at the VA based on our analysis – is central to the VA mission, and thus it is appropriate for the VA to invest in this area. In addition, the direction of research is driven by the interests and capabilities of the investigators that form the research community. It could be challenging for the VA to significantly change its research portfolio in the near term because only the individuals already employed at the VA are eligible for the research funding, limiting the pool of available expertise.

Furthermore, it might be appropriate for the VA to invest its funding in research on diseases that are not generously supported by other agencies, but which are prevalent in veterans. For example, the NIH is underfunding depression, lung cancer, injuries, chronic obstructive pulmonary disease, ischemic heart disease, and dementia [8]. We found that the volume of research on respiratory and cardiovascular diseases is lower than expected, based on health expenditures and limited NIH funding may provide an additional rational for the VA to increase its investments in research on these diseases.

Our approach has several limitations. First, demographic projections may be inaccurate, as many factors can change future trends. For example, the use of chemical weapons would alter the nature of war injuries; significant growth or decline in the job market would influence the number of veterans enrolled in the VA health care system; new or ongoing military commitments may increase the number of future veterans. Second, disease burden estimates did not include expenditures by veterans not enrolled in the VA health system or expenditures of enrolled veterans who received care outside of the VA. Third, we could not link approximately 15% of Principal Investigators and 17% of ICD-9 data to specific diseases or conditions. Finally, we classified complex research topics into broad disease groups and imposed a constraint of one disease per PI. This approach resulted in some misclassification of research volume.

While our study was done in the VA setting, other organizations can use this approach to plan and analyze their research portfolios. NIH employs a similar approach [15], and this method would be appropriate for other federal research funders as well as managed care organizations such as Kaiser Permanente, which combine provision of health care with research support.

In conclusion, the research portfolio at the VA is well aligned with health needs of veterans. The VA can continue to serve veterans by steering its research toward diseases and conditions which are expected to increase in the number of patients, costs of care, or both.
